# Activated Carbon as a Cathode for Water Disinfection through the Electro-Fenton Process

**DOI:** 10.3390/catal9070601

**Published:** 2019-07-12

**Authors:** Long Chen, Ameet Pinto, Akram N. Alshawabkeh

**Affiliations:** Department of Civil and Environmental Engineering, Northeastern University, Boston, MA 02115, USA

**Keywords:** activated carbon, electro-Fenton, hydrogen peroxide, water disinfection, *E. coli*

## Abstract

Unlike many other water disinfection methods, hydroxyl radicals (HO^•^) produced by the Fenton reaction (Fe^2+^/H_2_O_2_) can inactivate pathogens regardless of taxonomic identity of genetic potential and do not generate halogenated disinfection by-products. Hydrogen peroxide (H_2_O_2_) required for the process is typically electrogenerated using various carbonaceous materials as cathodes. However, high costs and necessary modifications to the cathodes still present a challenge to large-scale implementation. In this work, we use granular activated carbon (GAC) as a cathode to generate H_2_O_2_ for water disinfection through the electro-Fenton process. GAC is a low-cost amorphous carbon with abundant oxygen- and carbon-containing groups that are favored for oxygen reduction into H_2_O_2_. Results indicate that H_2_O_2_ production at the GAC cathode is higher with more GAC, lower pH, and smaller reactor volume. Through the addition of iron ions, the electrogenerated H_2_O_2_ is transformed into HO^•^ that efficiently inactivated model pathogen (*Escherichia coli*) under various water chemistry conditions. Chick–Watson modeling results further showed the strong lethality of produced HO^•^ from the electro-Fenton process. This inactivation coupled with high H_2_O_2_ yield, excellent reusability, and relatively low cost of GAC proves that GAC is a promising cathodic material for large-scale water disinfection.

## 1. Introduction

The burden of waterborne pathogens is significant and can lead to severe health complications [1,2]. To minimize the likelihood of contamination, drinking water is typically treated using a multi-barrier approach including disinfection and maintenance of a disinfectant residual during distribution of drinking water to the consumers. Most commonly used disinfection methods include ozonation, chlorination, chloramination, UV irradiation, and their combinations [3–5]. While each of these methods can be effective, they have key limitations. For instance, the electrochemical production of ozone relies on high voltage [6,7], and storage and transport of gaseous ozone is risky due to its high reactivity, thus it is usually produced onsite. Chlorination can result in formation of carcinogenic disinfection byproducts (DBP) [8], which may require pre-chlorination approaches to minimize DBP formation and additional significant efforts to manage post-chlorination DBP concentrations [9]. UV irradiation kills microorganisms by damaging double-stranded DNA [10,11], but this strategy is ineffective in turbid conditions; moreover, cells might reverse the DNA damage through a repair mechanism [12–14].

Hydroxyl radicals generated from H_2_O_2_ decomposition are effective in inactivating varying cell types, such as bacteria, eukaryotes, and viruses [15,16]. HO^•^ inactivates microbes primarily through the destruction of vital cellular components, including surface proteins, NAD(P)H, and DNA [17]. Post HO^•^ exposure, bacteria usually exhibit ruptured morphology [18], which indicates cell surface damage and osmotic shock due to leakage of cellular components. The severe destruction of cell structure is the most plausible reason for strong disinfection potential of HO^•^. Despite its effectiveness, the most significant challenge with the large-scale application of the Fenton process is the prohibitive cost of hydrogen peroxide (H_2_O_2_) (~USD $1.5/kg for bulk purchase) [19].

Recently, electrogeneration of H_2_O_2_ via two-electron reduction of oxygen has drawn considerable interest. With specific cathodes, dissolved oxygen accepts two electrons and converts to H_2_O_2_ through an oxygen reduction reaction [Equation (1)] [20,21]. Many types of carbon materials, such as reticulated vitreous carbon foam, graphite felt, carbon felt, and carbon nanotubes, have been used as cathodes [22]. Previous reports suggest that oxygen- and nitrogen-containing functional groups on the surface are particularly beneficial for electron transfer to oxygen [23]. Therefore, a large number of modification methods (e.g., acid/base incubation, pyrolysis of coated resins, etc.) to the abovementioned carbonaceous materials are being tested to enhance H_2_O_2_ titer [24,25]. However, these costly modifications are not ideally suitable for large-scale applications.

(1) $${\text{O}}_{2}+2H^{+}+2e^{-}\to H_{2}O_{2}(0.695\text{ V vs. SHE, standard hydrogen electrode})$$

Granular activated carbon (GAC) has long been used as an adsorbent to remove environmental contaminants [26]. It has been reported that electrochemical polarization, which creates an anode on one side and a cathode on the other side, could regenerate GAC adsorbent beds after saturated adsorption of organic contaminants [27,28]. During this process, H_2_O_2_ is generated on the cathode side, indicating that GAC has two-electron electrochemical oxygen reduction activity [28]. To date, studies using an entire GAC as a specific cathode to electrogenerate H_2_O_2_ are relatively lacking. Further, GAC is likely the cheapest functional carbon material, adding further viability towards its large-scale implementation in the future. In this study, we aim to evaluate the feasibility of using pristine GAC for water disinfection with *Escherichia coli* as a model pathogen. Electrogeneration of H_2_O_2_ was measured under various conditions, and HO^•^ from the reaction between iron ion and H_2_O_2_ was then used to inactivate *E. coli*. The development of the GAC-based electro-Fenton process for water disinfection is especially desirable in low-income regions and could meet the needs of their sustainable development. Besides, the novel application of amorphous GAC as a cathode has great implication in that other amorphous carbon materials (such as biochar manufactured from biomass precursor) could also be used for hydrogen peroxide generation. It is overall anticipated that this study could advance the application of low-cost carbons for electrochemical water treatment purpose.

## 2. Results and Discussion

### 2.1. Generation of Hydrogen Peroxide with GAC Cathode

The used GAC had a specific surface area of 840.5 m^2^/g, providing sufficient catalytic sites for H_2_O_2_ generation. Carbon- (C1s) and oxygen-containing (O1s) groups are abundant on the GAC surface [28] and increased the hydrophilicity and mediated the facile generation of H_2_O_2_ [23]. Typically, functional groups such as carboxyl and etheric groups are particularly favored for oxygen reduction into H_2_O_2_ [29]. We fabricated the cathode by loading a certain amount of GAC into a stainless-steel mesh. This hybrid cathode was tightly wrapped by a rubber band to ensure sufficient contact between the stainless-steel mesh and the conductive surface of GAC (Figure S1 of Supplementary Material). A Ti/MMO mesh anode was used to produce oxygen instead of air ventilation for H_2_O_2_ generation. The mechanism for H_2_O_2_ generation was initially explored in our previous work [28]. The GAC cathode produced a significant amount of H_2_O_2_ in the rage of 3–20 mg/L after 60 min (Table 1). This is particularly important, because other functional, higher priced carbonaceous materials typically produce 12–36 mg/L H_2_O_2_ in similar settings [30–32].

### 2.2. Parameters that Influenced H_2_O_2_ Yield

We tested various parameters that influenced the H_2_O_2_ yield (Table 1). Increasing GAC mass from 1 to 3 g in 200 mL solution increased H_2_O_2_ yield from 4.12 to 14.64 mg/L. The improved yield of H_2_O_2_ was primarily due to the increase in reactive sites with increasing GAC mass per electrolyte volume. Moreover, the electrogeneration of H_2_O_2_ by GAC cathode was more effective under acidic pH, as a higher concentration of protons could mediate a faster reaction rate. When the pH was reduced from 5 to 3, the H_2_O_2_ yield increased by 36.6% from 14.90 to 20.36 mg/L. However, the change of pH from 11 to 5 did not result in such drastic change in H_2_O_2_ production (i.e., 12.74 to 14.90 mg/L), because low proton concentration under this pH range limited H_2_O_2_ formation. We further tested H_2_O_2_ yield at different currents, while other parameters were fixed (3 g GAC, 200 mL volume, pH_ini_ 7). Increasing current from 50 to 100 mA increased H_2_O_2_ yield from 3.38 to 14.64 mg/L. Further increase in the current to 150 mA decreased H_2_O_2_ yield to 10.7 mg/L. This was presumably because of the parasitic H_2_O_2_ decomposition on the anode [28]. We assessed the effect of reactor volume, which is important for large-scale application in practice. Reaction conditions were 3 g GAC, 100 mA current, and pH_ini_ 7. Results showed that increasing reactor volume from 200 to 400 mL decreased the H_2_O_2_ yield from14.64 to 10.69 mg/L. The current efficiency, however, increased from 4.62% to 6.74%, indicating a higher accumulated H_2_O_2_ amount, primarily because the consumption pathway of H_2_O_2_ was weakened at low H_2_O_2_ concentration. These results suggest that larger reactor volume mediates greater utilization efficiency of the electrons.

The electrogeneration of H_2_O_2_ tended to acidify the solution, which was desirable for the Fenton reaction. This was because, during the electrogeneration of H_2_O_2_, the proton generation exceeded hydroxyl ion generation. As a result, solution pH was between 2.8 and 4.2 in assays after 60 min reaction (Table 1), and such acidity was suitable for the subsequent Fenton reaction.

It is important to note that the ability of GAC to generate H_2_O_2_ is feasible under various granularities. We tested GAC of varying sizes (i.e., 4–8 mesh, 4–12 mesh, 4–14 mesh, and 5 mm) and found that they consistently produced H_2_O_2_ with a yield of 11–16 mg/L, exhibiting approximately similar oxygen reduction activities. This was likely because oxygen reduction to H_2_O_2_ occurred on the catalytic sites on both internal pores and the external surface, and the GAC granularity thus did not affect the total number of catalytic sites. This indicated that the electrogeneration of H_2_O_2_ could occur on a variety of GAC types, suggesting the practical applicability of this method.

### 2.3. Disinfection of E. coli through Electro-Fenton Process

In this study, electrogenerated H_2_O_2_ was used for water disinfection of *E. coli* as a model pathogen. The disinfection efficiency of H_2_O_2_ alone is relatively low, because the genetically encoded catalases can efficiently decompose H_2_O_2_ into water and oxygen [33]. In contrast, HO^•^ from the Fenton reaction could effectively kill a wide variety of microorganisms regardless of their cellular structure or genetic potential [15,16].

**Figure 1.**
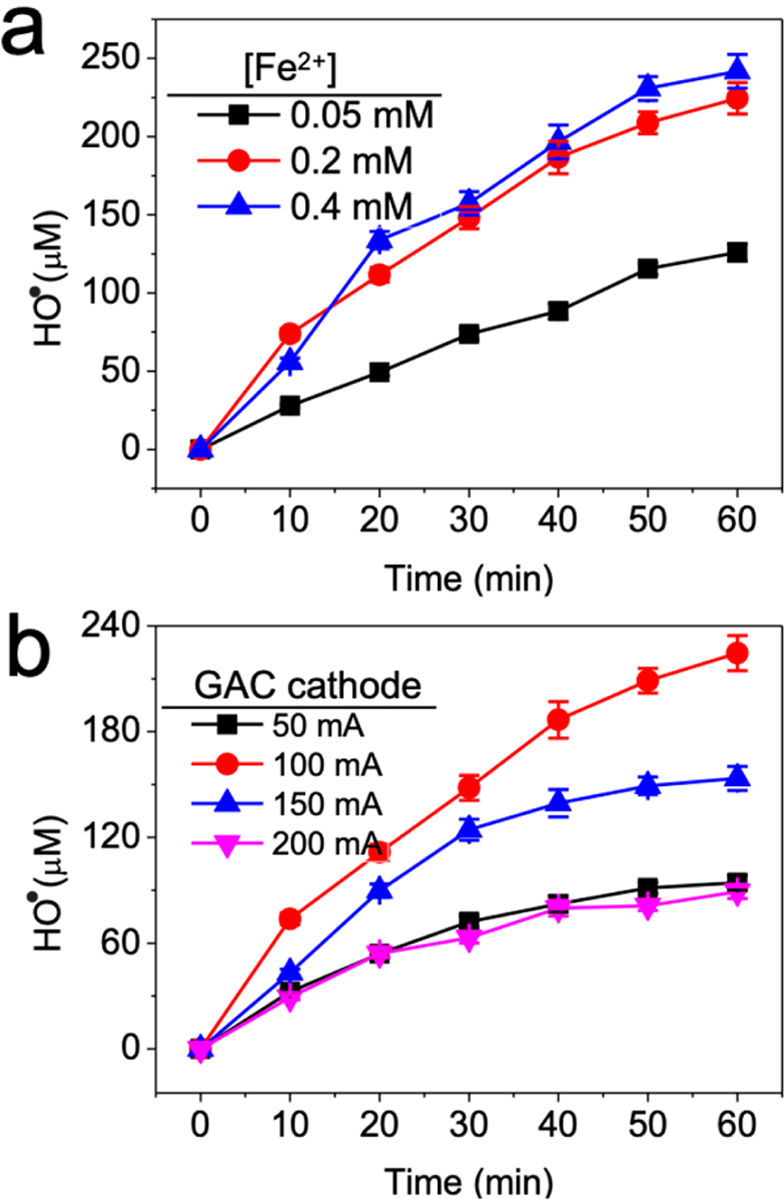
Impact of (**a**) ferrous iron ion concentration and (**b**) GAC cathode current intensity on hydroxyl radical generation during the electro-Fenton process. Conditions: (**a**) 3 g GAC as cathode under 100 mA current, pH_ini_ 7; (**b**) 3 g GAC as cathode, 0.2 mM FeSO_4_, pH_ini_ 7.

Electrogenerated H_2_O_2_ was transformed into highly oxidizing HO^•^ in the presence of ferrous iron ions. The process of electrogeneration of H_2_O_2_ from the GAC cathode and transformation into HO^•^ by iron ions is known as the electro-Fenton process. The HO^•^ yield was measured with the benzoic acid quantification method [34]. Iron ions concentrations of 0.05, 0.2, and 0.4 mM resulted in the production of 125.9, 224.53, and 241.8 μM HO^•^ after 60 min of the electro-Fenton process, respectively (Figure 1a), with no significant difference in HO^•^ generated between 0.2 mM and 0.4 mM concentrations. Moreover, the GAC cathode at 100 mA mediated the highest HO^•^ yield compared with other electric currents (Figure 1b), indicating that there is an optimum value for the current.

#### 2.3.1. Mechanism of Electro-Fenton Disinfection

**Figure 2.**
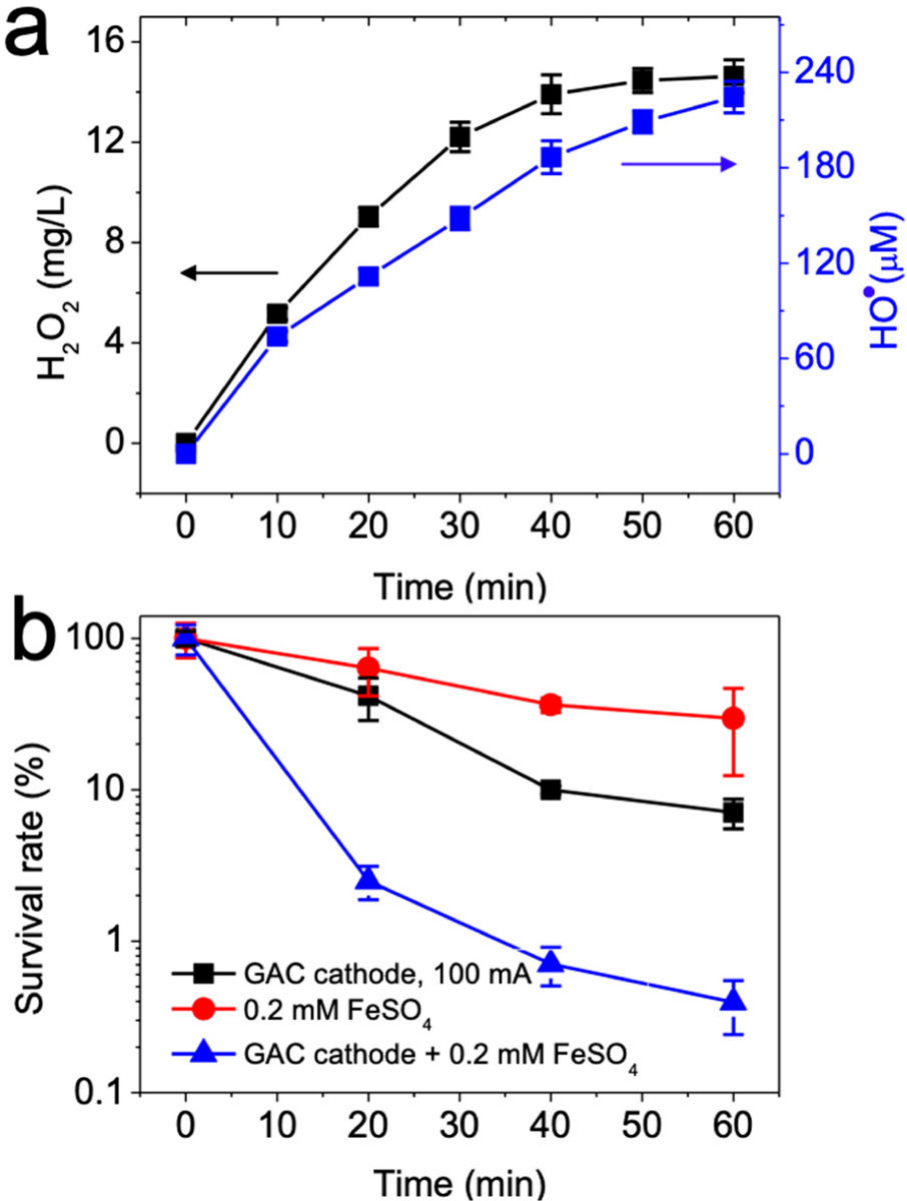
(**a**) GAC as the cathode to produce hydrogen peroxide and hydroxyl radical after addition of iron ions and (**b**) generated hydroxyl radical inactivated *E. coli*. Conditions: 3 g GAC as cathode under 100 mA current, 0.2 mM FeSO_4_, 10^8^ CFU/mL (CFU, colony forming unit) *E. coli*, pH_ini_ 7.

Treatment by either GAC cathode or ferrous ions alone mediated 1.15- and 0.53-log reduction in cell viability, respectively. The electro-Fenton process coupling both GAC cathode and ferrous ions led to a 2.40-log inactivation of *E. coli* cells, as the generated HO^•^ was a strong bactericide (Figure 2a,b). The GAC cathode under 100 mA inactivated *E. coli* mainly via the combination of generated H_2_O_2_ and acidification (pH decreased to 3.45, Table 1), whereas H_2_O_2_ at 2 mM or acid pH of 3 individually led to less than 0.46-log of bacterial inactivation (Figure S3 of Supplementary Material). H_2_O_2_ slightly inactivated bacteria, likely through the oxidation of sulfhydryl groups of functional enzymes [35]. Acidic pH denatured the protein structure and may also have increased the permeability of the cell membrane [36]. In addition, ferrous ions were slightly bactericidal after oxidation into ferric ions (Fe^3+^), which could have denatured the functional enzymes through the oxidation of side chains of amino acids [37].

**Figure 3.**
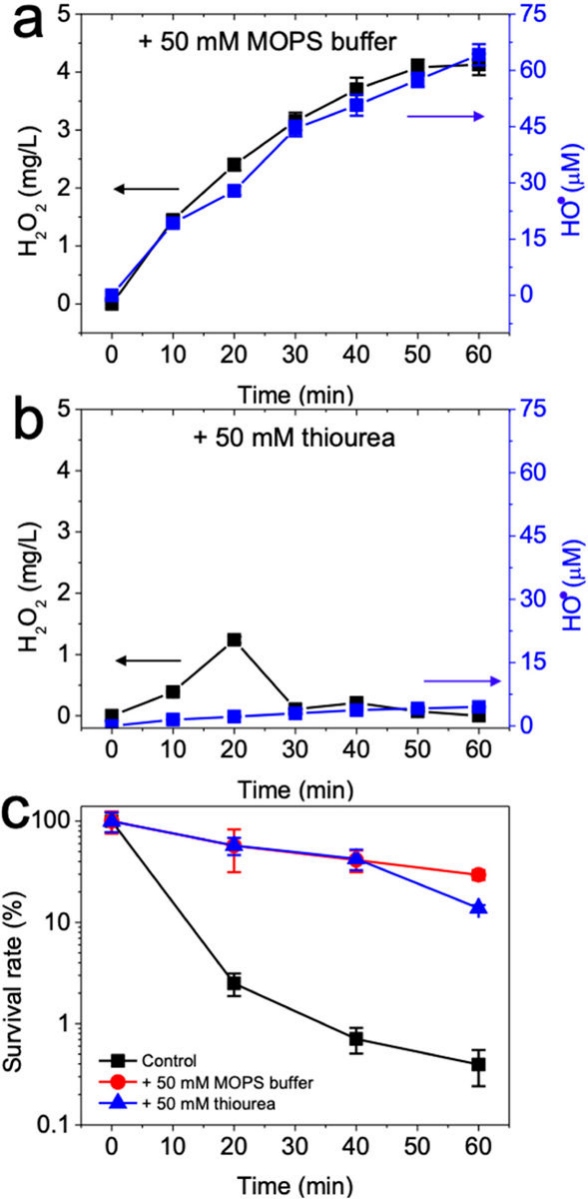
Yields of hydrogen peroxide and hydroxyl radical during electro-Fenton process after addition of (**a**) 50 mM MOPS buffer or (**b**) 50 mM thiourea and (**c**) performance of bacterial disinfection performance. Conditions: 3 g GAC as cathode under 100 mA current, 0.2 mM FeSO_4_, 10^8^ CFU/mL *E. coli*, pH_ini_ 7. Control indicates electro-Fenton process.

Nonetheless, HO^•^ likely played a major role in *E. coli* inactivation. Addition of 50 mM MOPS buffer (3-(N-morpholino)propanesulfonic acid) to attenuate solution acidification of the electro-Fenton process (Figure 3a) decreased H_2_O_2_ yield from 14.64 to 4.13 mg/L, using the GAC as a cathode after a 60 min reaction. Consistently, HO^•^ yield was also reduced from 224.53 to 64.15 μM in the electro-Fenton process. Furthermore, the addition of 50 mM MOPS buffer to the electro-Fenton process resulted in 0.53-log *E. coli* cell inactivation due to reduced HO^•^ production (Figure 3c), which was significantly less than the electro-Fenton disinfection without the buffer (i.e., 2.40-log). The use of 50 mM thiourea to quench H_2_O_2_ and HO^•^ (Figure 3b) resulted in negligible concentrations of H_2_O_2_ or HO^•^ during the course of the electro-Fenton reactions, and concomitant decrease in the inactivation of *E. coli* cells (1.11-log) was observed after 60 min (Figure 3c). Taking these results together, we concluded that the disinfection of *E. coli* by the electro-Fenton process was primarily due to the HO^•^ from electrogenerated H_2_O_2_ under activation of ferrous ions in an acidic pH.

#### 2.3.2. Complete Disinfection of *E. coli* of Various Concentrations

**Figure 4.**
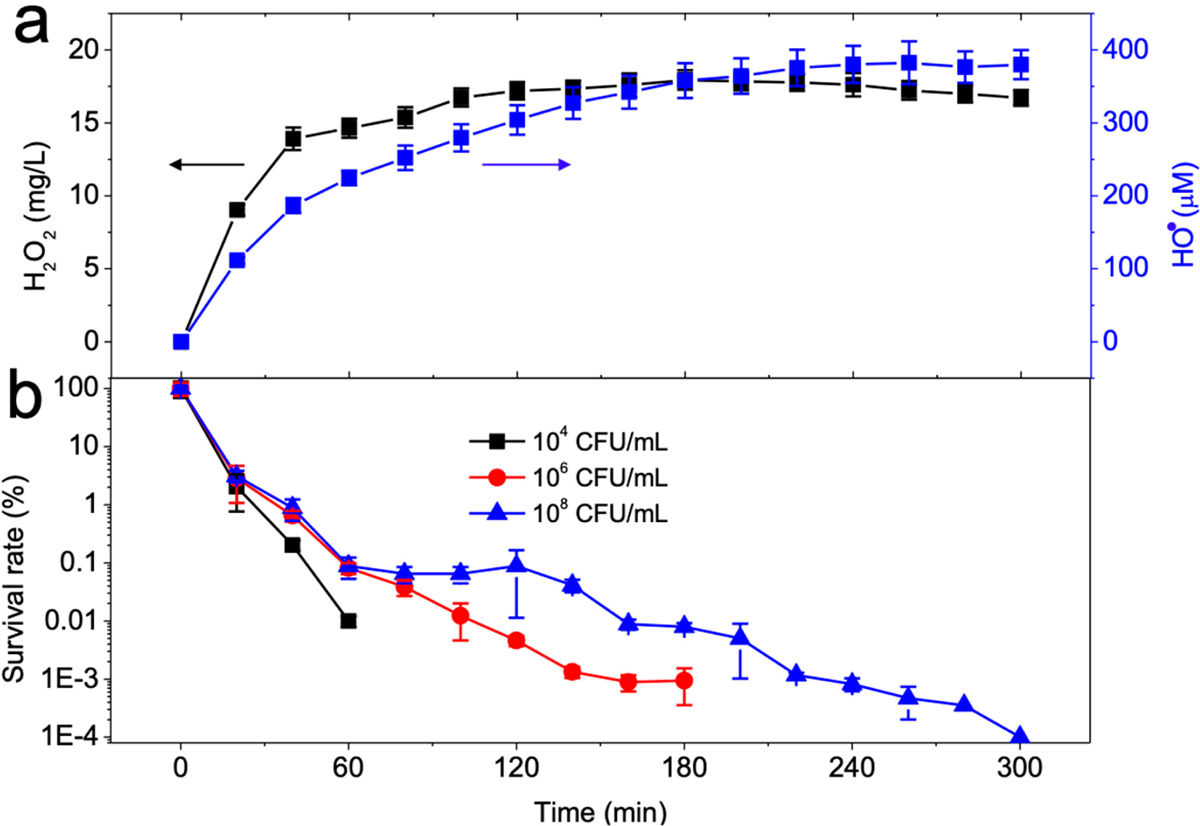
(**a**) Yields of hydrogen peroxide and hydroxyl radical by electro-Fenton process during 300 min reaction and (**b**) bacterial disinfection performances at different bacteria concentrations. Conditions: 3 g GAC as cathode under 100 mA current, 0.2 mM FeSO_4_, 10^8^ CFU/mL *E. coli*, pH_ini_ 7. *E. coli* concentration in (**b**) varied as indicated.

Continuous monitoring of H_2_O_2_ and HO^•^ yields over 300 min (Figure 4a) revealed that H_2_O_2_ generation was rapid during the first 40 min then slowed down. H_2_O_2_ concentration reached a plateau of 16.74 mg/L at 100 min. The measured H_2_O_2_ concentration was the total H_2_O_2_ generation minus H_2_O_2_ decomposition [Equation (10)]. Total electrogeneration rate of H_2_O_2_ was determined by applied electric potential and dissolved oxygen content, while H_2_O_2_ decomposition was largely affected by H_2_O_2_ concentration. Therefore, as the H_2_O_2_ concentration in bulk solution increased, its decomposition rate increased due to the more prominent parasitic pathways [Equations (3)–(6)]. When the H_2_O_2_ generation rate was equal to the decomposition rate, an equilibrium concentration (plateau) was reached in agreement with electrogeneration of H_2_O_2_ on other cathodes [30,32].

(2) $${\left[H_{2}O_{2}\right]}_{\text{measured}}=\int_{0}^{t}r_{{\left[H_{2}O_{2}\right]}_{\text{generation}}}dt-\int_{0}^{t}r_{{\left[H_{2}O_{2}\right]}_{\text{decomposition}}}dt$$

Parasitic H_2_O_2_ decomposition pathways:

(3) $$H_{2}O_{2}+2H^{+}+2e^{-}\to 2H_{2}O$$

(4) $$H_{2}O_{2}\to HO_{2}^{•}+H^{+}+e^{-}$$

(5) $$HO_{2}^{•}\to O_{2}+H^{+}+e^{-}$$

(6) $$2H_{2}O_{2}\to H_{2}O+O_{2}$$

We added 0.2 mM ferrous ions into the solution to transform H_2_O_2_ into HO^•^. The generated Fe^3+^ after the reaction could then be reduced into Fe^2+^ on the cathode and again used for H_2_O_2_ activation. Hence, the HO^•^ was constantly generated in the presence of both the GAC cathode and the iron ion, as quantified via the addition of benzoic acid (Figure 4a). The HO^•^ yield at 100 min was 279.61 μM and reached 379.84 μM after 300 min.

The disinfection of *E. coli* at 10^4^, 10^6^, and 10^8^ CFU/mL was tested respectively over 300 min (Figure 4b). The electro-Fenton process could completely remove 10^4^ CFU/mL *E. coli* within 60 min, and 5.02-log *E. coli* removal was achieved after 180 min at an initial concentration of 10^6^ CFU/mL. The process also inactivated 6.1-log *E. coli* after 300 min with an initial concentration of 10^8^ CFU/mL. The results indicate that GAC as a cathode could efficiently inactivate *E. coli* at varying concentrations.

#### 2.3.3. Chick–Watson Model

**Figure 5.**
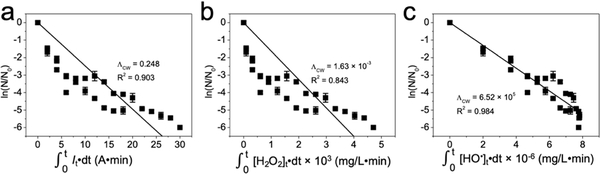
Chick–Watson model fitting with (**a**) electric current, (**b**) generated hydrogen peroxide, and (**c**) produced hydroxyl radical as input disinfectant.

*E. coli* inactivation kinetics was fitted with the first-order Chick–Watson model. CT values (disinfectant concentration × contact time) for electric current, H_2_O_2_, and HO^•^ as input disinfectant were individually plotted against decimal logarithm of bacterial survival rate [i.e., ln(N/N_0_)] (Figure 5). The Chick–Watson coefficients (Λ_CW_) for electric current, H_2_O_2_, and HO^•^ were determined to be 0.248 A^−1^ min^−1^, 1.63 × 10^−3^ mg^−1^ L min^−1^, and 6.52 × 10^5^ mg^−1^ L min^−1^, respectively. Chick–Watson model fitting with HO^•^ as disinfectant showed the highest correlation coefficient (R^2^ = 0.984) compared with fitting results of electric current (R^2^ = 0.903) and H_2_O_2_ (R^2^ = 0.843), presumably because HO^•^ is the species directly responsible for *E. coli* inactivation. Interestingly, the Λ_CW_ of HO^•^ in this study was 3.9 times greater than the reported value under neutral conditions (1.33 × 10^5^ mg^−1^ L min^−1^) [38], possibly because other parameters such as acidic conditions synergistically enhanced the bactericidal role of HO^•^. Moreover, the lethality of HO^•^ (2.70 V) was several orders higher than ozone (50 mg^−1^ L min^−1^, 2.07 V), chlorine (15.4 mg^−1^ L min^−1^, 1.36 V), or chlorine dioxide (25 mg^−1^ L min^−1^, 1.91 V), primarily due to its strong oxidation potential [38].

### 2.4. Resistance to Water Alkalinity

The generation of HO^•^ radical bactericide from the Fenton reaction relies on an acidic pH. However, most natural water bodies can maintain buffering capacity, which is called water alkalinity, because of carbonate ions from atmospheric carbon dioxide or sediment rock mineral. The typical alkalinity of waters is below 200 mg CaCO_3_/L [39,40]. Thus, large amounts of acid are necessary to overcome such high alkalinity, or the efficiency of the traditional Fenton process for water treatment would be severely compromised. Given that produced protons on the anode exceed released hydroxyl ions on the cathode, the electro-generation of H_2_O_2_ led to an automatic acidification of the solution, which facilitated the Fenton reaction. However, little is known about solution acidification by the electrogeneration of H_2_O_2_ in carbonated media.

**Figure 6.**
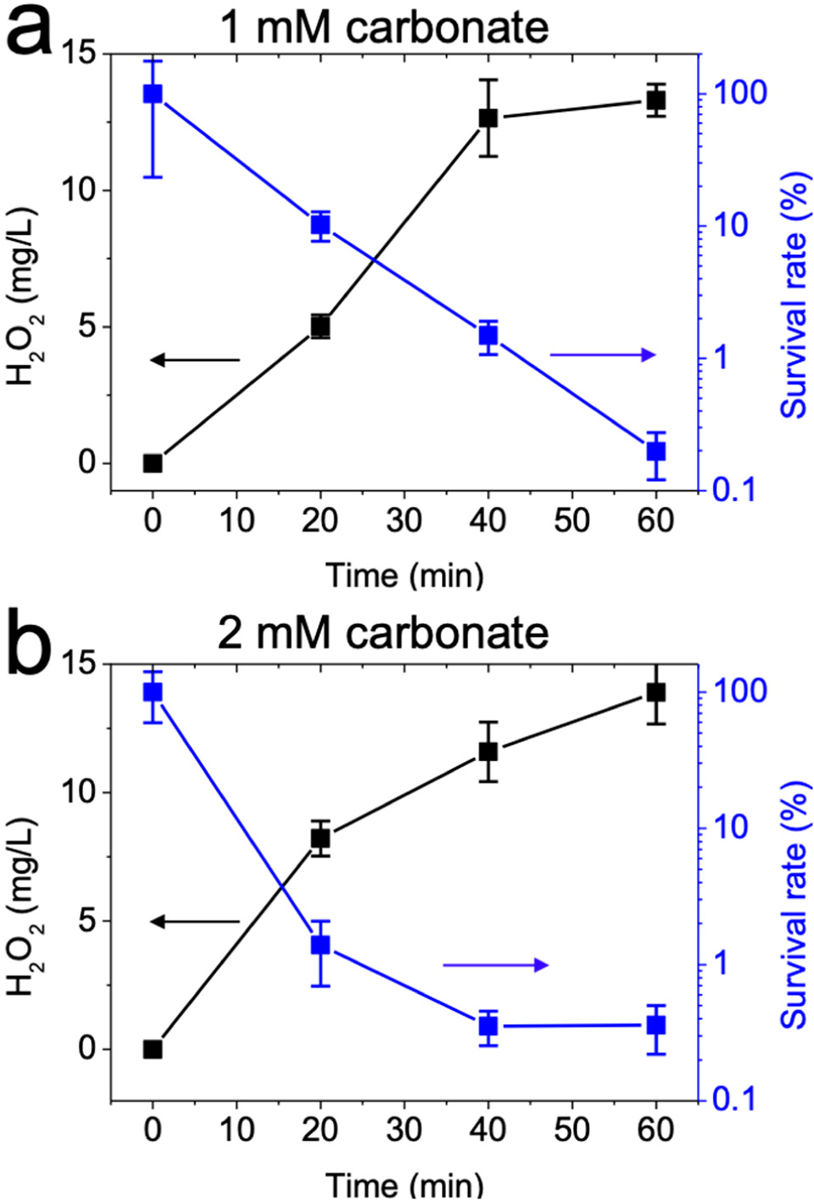
Effect of carbonate on hydrogen peroxide production or bacterial disinfection at (**a**) 1 mM or (**b**) 2 mM. Conditions: 3 g GAC as cathode under 100 mA current, 0.2 mM FeSO_4_, 10^8^ CFU/mL *E. coli*. Carbonate concentration was indicated. Reaction solution was adjusted to pH 7 after addition of carbonate ions.

The effectiveness of the electro-Fenton process toward *E. coli* disinfection was therefore tested under two representative alkalinity conditions. It was observed that electrogeneration of H_2_O_2_ tended to acidify the solution regardless of carbonate concentration, and solution pH decreased to 3.45 in the presence of 2 mM carbonate after 60 min. Moreover, the presence of 1–2 mM carbonate ion exhibited negligible impact on H_2_O_2_ generation (Figure 6). Specifically, the electrogenerated H_2_O_2_ yields were 14.64, 13.31, and 13.91 mg/L by GAC cathode with 0, 1, and 2 mM carbonate, respectively. As a result, the disinfection efficiency by the electro-Fenton process was not affected by the presence of carbonate ions. This further highlights the practicality of the electro-Fenton process for water disinfection under buffered conditions.

### 2.5. Reusability of GAC Cathode

The active sites on the GAC surface are under cathodic potential during the electro-Fenton process. This could result in the reductive elimination of functional groups of certain carbonaceous materials, and the potency to produce H_2_O_2_ might drastically decrease [30]. GAC is known to harbor abundant functional groups that exist both on the external surface and the internal pore surface [41]. Thus, it is important to assess the effect of long-term cathodic current-mediated oxygen reduction on the catalytic activity of GAC.

**Figure 7.**
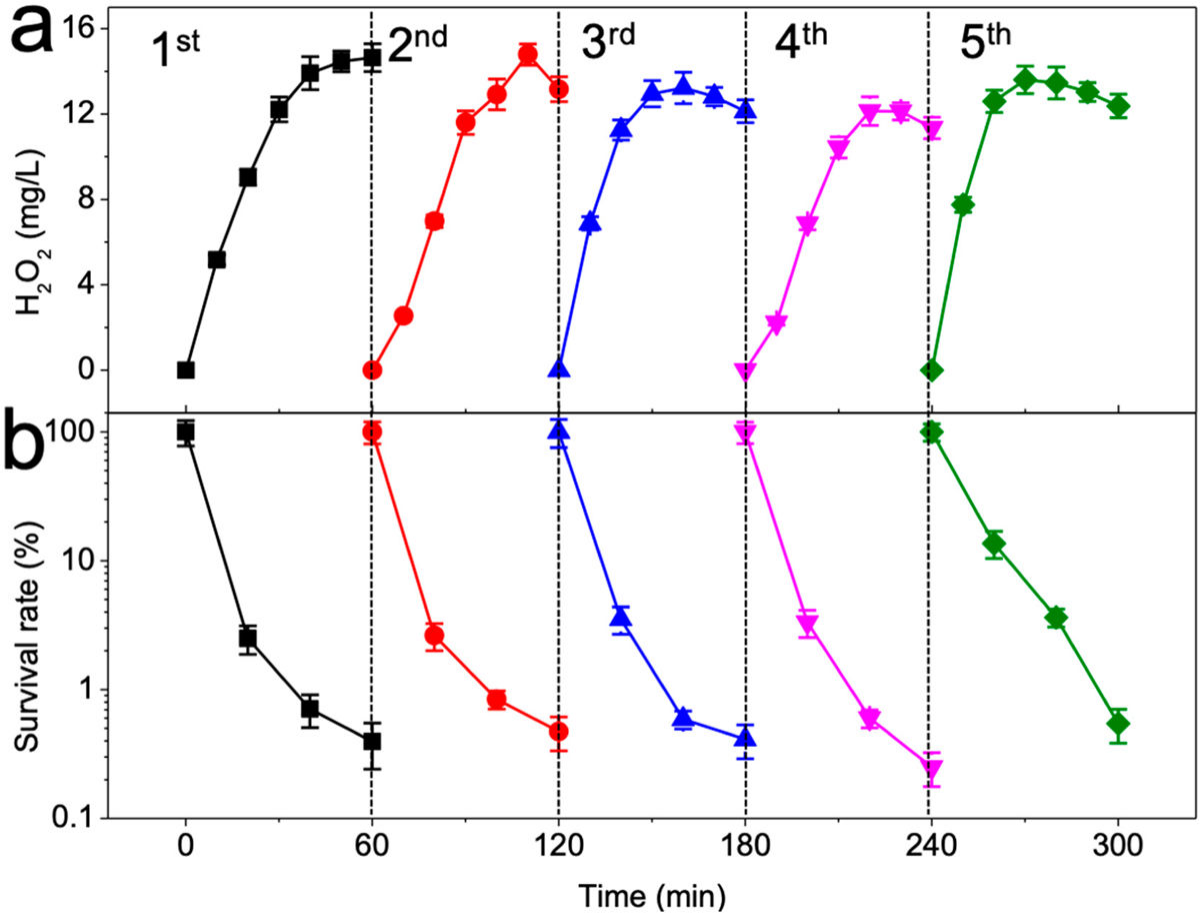
Reusability of GAC cathode for (**a**) hydrogen peroxide production and (**b**) bacterial disinfection. Conditions: 3 g GAC as cathode under 100 mA current, 0.2 mM FeSO_4_, 10^8^ CFU/mL *E. coli*, pH_ini_ 7.

We tested the durability of the GAC cathode for multiple rounds of application for water disinfection. After each round of the electro-Fenton reaction, the GAC was extensively washed with diluted sulfuric acid and then rinsed with Milli-Q water. The cleaned and oven-dried GAC was then applied to the next round of the electro-Fenton reaction. The H_2_O_2_ yields were sustained between 11.34 and 14.64 mg/L without notable decrease for a minimum of five rounds of application and regeneration (Figure 7a). Moreover, with the addition of ferrous ions, the stable H_2_O_2_ generation consistently led to 2.26–2.60 log reduction of *E. coli* in each round due to the production of HO^•^ radicals (Figure 7b).

### 2.6. Disinfection of E. coli with Antibiotic-Resistance Genes

Bacteria hosting antibiotic-resistance genes (ARGs) have been an emerging concern in water treatment, and they are resistant to common antibiotic drugs due to inherent efflux pumps [42]. In addition, ARGs of a bacterial species could be relayed to another bacterial species through horizontal gene transfer [43]. Previous research reported that, among all the water disinfection technologies, only advanced oxidation processes generating oxidative radicals (such as HO^•^) could potently damage ARGs and effectively prevent horizontal gene transfer [44].

**Figure 8.**
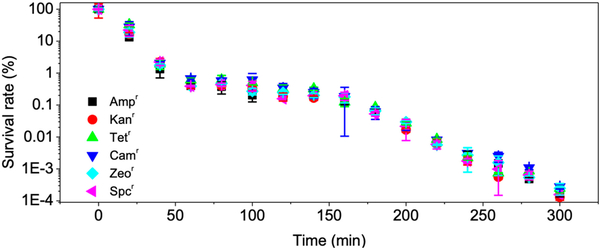
Electro-Fenton process disinfection performance towards *E. coli* of different antibiotic resistances. Conditions: 3 g GAC as cathode under 100 mA current, 0.2 mM FeSO_4_, 10^8^ CFU/mL *E. coli*, pH_ini_ 7. Amp^r^, ampicillin-resistant; Kan^r^, kanamycin-resistant; Tet^r^, tetracycline-resistant; Cam^r^, chloramphenicol-resistant; Zeo^r^, zeocyin-resistant; Spc^r^, spectinomycin-resistant.

Electrogeneration of H_2_O_2_ with the GAC cathode is a practical method and is affordable by most water treatment plants. We utilized the electro-Fenton process to disinfect *E. coli* with various ARGs. Antibiotic-resistant *E. coli* cells were obtained after transferring plasmids encoding corresponding ARGs into competent cells followed by antibiotic selection on a Luria-Bertani (LB)-agar plate. Results indicated that the electro-Fenton process could non-selectively inactivate *E. coli* with different ARGs (e.g., ampicillin-, kanamycin-, tetracyclin-, chloramphenicol-, zeocin-, and spectinomycin-resistance genes). For instance, 2.18–2.41 log of *E. coli* cells were reduced after 1 h treatment (Figure S4 of Supplementary Material). Additionally, the complete disinfections of *E. coli* cells were obtained after 300 min (Figure 8) regardless of the hosted ARGs, highlighting the great promise of the process. However, this was not surprising, because HO^•^ disinfection mainly occurs through destruction of cellular structure and is not related to genetic potential.

### 2.7. Techno-Economic Analysis

GAC as a porous carbon material has long been used as an adsorbent for the removal of water contaminants. Owing to the various functional groups on the surface, the GAC cathode is reported to mediate hydrogen peroxide formation via an oxygen reduction reaction. An important advantage of the GAC cathode is the relatively low cost. The market price of GAC is around USD $0.75 per kilogram in a large-scale purchase [45]. Moreover, GAC can be reused for multiple rounds without loss of catalytic activity, further lowering the operation cost. GAC can also be produced from the pyrolysis of biomass, such as coconut shell [46] or other waste materials [47]. The utilization of waste biomass to generate H_2_O_2_ for water treatment is especially attractive, considering that both water treatment and biomass treatment consume large amounts of cost and energy. The application of GAC as a cathode for water disinfection is thereby promising.

Unlike traditional water treatments, which rely on a single dose of a high concentration of oxidants, the electro-Fenton process continuously generates a low concentration of H_2_O_2_. The low concentration of H_2_O_2_ is instantly converted into HO^•^ in the presence of ferrous ions. In fact, the continuous and mild generation of oxidants is more favored than the single dose of high concentration of oxidants in practice, because the former method holds great promise to address the contaminants rebound phenomena [48,49]. Besides the contaminants rebound, residual water pathogens after a single treatment can also proliferate with a supply of nutrients. The developed electro-Fenton process is anticipated to completely eliminate water pathogens with continuous generation of H_2_O_2_.

## 3. Materials and Methods

### 3.1. Materials

Granular activated carbon (GAC, 4–8 mesh) as a cathode for oxygen reduction was obtained from Calgon Carbon Corporation. GACs of other sizes, i.e., 4–12 mesh, 4–14 mesh, and < 5 mm, were obtained from Calgon Carbon Corporation as well. Sodium sulfate (Na_2_SO_4_, ≥99%), titanium sulfate (TiSO_4_, 99.9%), ferrous sulfate (FeSO_4_), hydrogen peroxide (30% wt.), sodium carbonate (Na_2_CO_3_), and benzoic acid (C_7_H_6_O_2_, >99%) were purchased from Fisher Scientific (Hampon, NH, USA). MOPS buffer (C_7_H_15_NO_4_S) was purchased from Corning Inc. (Corning, NY, USA). Thiourea (CH_4_N_2_S, >99%) as a quencher of H_2_O_2_ and HO^•^ was purchased from ACROS Organics Inc. Luria-Bertani (LB) broth and agar for *E. coli* enumeration were purchased from BD, Difco Inc. Meshed Ti/mixed metal oxide (Ti/MMO, 3N International) was used as anode material.

### 3.2. Characterizations of Granular Activated Carbon

Nitrogen adsorption/desorption isotherm of the employed GAC was measured at −196 °C (ASAP 2420 V2.05). Then, the Brunauere–Emmete–Teller (BET) specific surface area was calculated from the isotherm curve. The volumes of micropore and mesopore were estimated using the Horvath–Kawazoe method and the Barrett–Joynerand–Halenda method, respectively. The pore size distribution was determined using the nonlocal density functional theory by the adsorption branch, and the total pore volume was calculated from the nitrogen amount adsorbed at a relative pressure of 0.975. Results showed that the GAC was microporous with an average pore width of less than 1 nm, and the specific surface area was 840.5 m^2^/g.

### 3.3. Batch Experiments for Disinfection

Before the experiments, 10 g GAC was pre-treated by vigorously stirring in 300 mL Milli-Q water with a magnetic bar for 10 min. The eluent was discarded, and GAC pellets were re-washed with 300 mL Milli-Q water. The washing steps were repeated until the eluent was clear. The GAC particles were then dried at 80 °C for 2–3 h. Log growth phase *E. coli* culture [200 mL, 10^8^ CFU/mL (CFU, colony forming unit)] was used for electro-Fenton disinfection experiments. Specifically, a single colony of *E. coli* K12 from an LB-agar plate was seeded in LB nutrient broth and cultured at 37 °C overnight. Then, 1% of the fully-grown bacteria solution was diluted in a sterile flask containing fresh LB medium and cultured for around 2 h until the log phase (OD_600_ ~ 0.8–1). Bacteria were harvested by centrifuging the *E. coli* culture at 10,000 g for 10 min, followed by intensive washing with Milli-Q water. The centrifugation and the washing steps were repeated three times, and the washed *E. coli* culture was used for experiments. During the electro-Fenton process, 3 g GAC held in a stainless-steel mesh was used as a cathode under 100 mA current with 10 mM Na_2_SO_4_ electrolyte and 0.2 mM FeSO_4_ as the catalyst. A schematic configuration of the reactor is shown in Figure S1 of Supplementary Material. Every 20 min, the bacterial solution was withdrawn for subsequent quantification.

### 3.4. E. coli Quantification Method

*E. coli* was quantified through a serial dilution method as reported previously [50–52]. Briefly, the sampled *E. coli* solution was put in the first row of a sterilized 96-well plate. The ten-fold serial dilution was performed by taking 20 μL well-mixed bacterial solution into the next row containing 180 μL phosphate buffered saline (PBS) physiological buffer. A total of 5 μL liquid from the serial dilution wells was plated on the LB-agar plate and incubated at 37 °C overnight. The survival rate was calculated from the CFU after treatment divided by that before treatment [Equation (7)].

(7) $$\text{Survival rate }=\frac{\text{ bacteria after treatment }(\text{CFU}/\text{mL})}{\text{ bacteria before treatment }(\text{CFU}/\text{mL})}\times {\text{log}}_{10}10$$

### 3.5. Hydrogen Peroxide and Hydroxyl Radical Quantification Method

The generated H_2_O_2_ using the GAC cathode without iron ions was quantified by complexing with titanium sulfate [53]. The absorbance of the developed yellow color was measured with a UV-Vis spectrometer at 405 nm. H_2_O_2_ generation was quantified under various conditions, e.g., GAC amount, solution pH, reactor volume, GAC granularity, and current intensity. During the electro-Fenton process, HO^•^ was generated from the Fenton (Fe^2+^/H_2_O_2_) reaction using the GAC cathode in the presence of iron ions and quantified using benzoic acid. Three grams of GAC hosted by stainless-steel mesh were transferred into a 200 mL solution of 10 mM Na_2_SO_4_ and 10 mM benzoic acid at pH 7 overnight under constant stirring. Benzoic acid saturated GAC was then used as a cathode under 100 mA for the electro-Fenton reaction with the addition of 0.2 mM FeSO_4_. The primary products from HO^•^ oxidizing benzoic acid were mainly o-, m-, and p-hydroxybenzoic acid, and the stoichiometric ratio of HO^•^ to produced p-hydroxybenzoic acid was determined to be 5.87 ± 0.18 [34]. Hence, HO^•^ yield was calculated from p-hydroxybenzoic acid concentration multiplied by 5.87. p-Hydroxybenzoic acid concentration was measured with a high-performance liquid chromatography (HPLC, Agilent 1200 Infinity Series) equipped with an Agilent Eclipse AAA C18 column (4.6 × 150 mm) with 0.5 mL/min methanol/1% phosphoric acid (20/80) used as the mobile phase and p-hydroxybenzoic acid with a retention time of 5 min and was detected at 210 nm wavelength using an Agilent 1260 diode array detector.

### 3.6. Current Efficiency Calculation

The current efficiency (CE) for H_2_O_2_ generation, defined as the ratio of the electrons consumed by the two-electron oxygen reduction reaction over the total electrons passing through the circuit, was calculated by Equation (8).

(8) $$CE=\frac{nFC_{H_{2}O_{2}}V}{\int_{0}^{t}Idt}\times 100\%$$

where *n* is the number of electrons transferred for O_2_ reduction to H_2_O_2_, *F* is the Faraday constant (96486 C/mol), *C*_*H2O2*_ is the concentration of H_2_O_2_ (mol/L), *V* is the electrolyte volume (L), *I* (A) is the current at time *t*, and *t* is the reaction time (s).

### 3.7. Reusability Assay of Granular Activated Carbon

After each round of the electro-Fenton reaction, GAC was washed with 0.1 M H_2_SO_4_ acid solution to dissolve iron precipitates under vigorous stirring for 3 h. The cleaned GAC particles were then collected and rinsed with Milli-Q water to remove residual acid until eluent liquid had neutral pH. The regenerated GAC was further dried in an oven at 80 °C for 2–3 h prior to utilization as a cathode for the subsequent electro-Fenton process.

### 3.8. Disinfection of Antibiotic-Resistant Bacteria

*E. coli* K12 strains harboring ampicillin-, kanamycin-, tetracycline-, chloramphenicol-, zeocin-, and spectinomycin-resistance biomarkers were obtained by transferring pET-32a(+), pET24a(+), pTet, pCam, pCC-zeo, and pCC-spc plasmids (Figure S2 of Supplementary Material), respectively, into *E. coli* K12 competent cells. The bacteria with successful transfers of the biomarker gene were selected on an LB-agar plate with corresponding antibiotics (i.e., 30 μg/mL ampicillin, 50 μg/mL kanamycin, 20 μg/mL tetracycline, 20 μg/mL chloramphenicol, 25 μg/mL zeocin, and 100 μg/mL spectinomycin). Then, 10^8^ CFU/mL of various antibiotic-resistant *E. coli* cells in the log phase were disinfected by the electro-Fenton process using GAC cathode under 100 mA with 10 mM Na_2_SO_4_ electrolyte and 0.2 mM FeSO_4_ at pH 7.

### 3.9. Chick–Watson Model

In the Chick–Watson model, the bacterial disinfection efficiency (ln(N/N_0_)) is correlated with both disinfectant concentration (C) and contact time (T), which can be expressed as:

(9) $$\text{ln}\left(\text{N}/{\text{N}}_{0}\right)=\Lambda_{\text{CW}}\text{CT}$$

where Λ_CW_ is the Chick–Watson coefficient. Electric current, generated H_2_O_2_, and produced HO^•^ as input disinfectants were investigated individually. Specifically, CT value for electric current was 0.1 t (A•min) under a constant 100 mA current.

For CT value of generated H_2_O_2_:

(10) $$\int_{0}^{t}{\left[H_{2}O_{2}\right]}_{t}dt\approx \sum_{0}^{t}0.5\left({\left[H_{2}O_{2}\right]}_{t}+{\left[H_{2}O_{2}\right]}_{t+\Delta t}\right)\Delta t$$

For CT value of produced HO^•^:

(11) $$\int_{0}^{t}{\left[{\text{HO}}^{•}\right]}_{t}dt\approx \sum_{0}^{t}0.5\left({\left[{\text{HO}}^{•}\right]}_{t}+{\left[{\text{HO}}^{•}\right]}_{t+\Delta t}\right)\Delta t$$

During disinfection experiments, the steady-state HO^•^ concentration was estimated using a similar approach to Cho et al. [38] by adding 0.5 mM benzoic acid as a probe compound. The decay of benzoic acid obeyed the following equations:

(12) $${\text{HO}}^{•}+\text{ benzoic acid }\to \text{ products }$$

(13) $$-\frac{d[\text{benzoic acid}]}{dt}=k_{7}\left[{\text{HO}}^{•}\right][\text{benzoic acid}]$$

(14) $${\left[{\text{HO}}^{•}\right]}_{t}=-\frac{d[\text{benzoic acid}]}{k_{7}[\text{benzoic acid}]dt}\approx -\frac{2\Delta [\text{benzoic acid}]}{k_{7}\left({\left[\text{benzoic acid}\right]}_{t}+{\left[\text{benzoic acid}\right]}_{t+\Delta t}\right)\Delta t}$$

where k_7_ is 1.8 × 10^9^ M^−1^ s^−1^ [54].

## 4. Conclusions

In this work, we used activated carbon as a cathode to generate H_2_O_2_ for the purpose of water disinfection. Ferrous ions were added to transform H_2_O_2_ into strong bactericide HO^•^. Parameters including GAC loading, currents, solution pH, and reactor volume were investigated. The highest concentration of H_2_O_2_ reached 20.36 mg/L under optimal conditions. With ferrous ions, H_2_O_2_ was transformed into HO^•^ to inactivate *E. coli*. This electro-Fenton process could inactivate 10^8^ CFU/mL *E. coli* after 300 min regardless of bacterial ARGs. Moreover, the electro-Fenton process automatically acidified the solution and was resistant to a high concentration of buffering carbonates in water. Considering the cost-effectiveness of GAC, this electro-Fenton process is promising in practical water treatment applications. Future work includes the upscaling of this process to test its feasibility in large-scale practice.

## Supplementary Material

supple

## Figures and Tables

**Table 1.** Yields of hydrogen peroxide and determined current efficiencies after reaction for 60 min under various conditions.

Granular Activated Carbon (GAC) Amount (g)	pH_ini_	Current (mA)	Volume (mL)	Granularity (mesh)	pH_final_	H_2_O_2_ Yield (mg/L)	Current Efficiency
1	7	100	200	4–8	3.54 ± 0.16	4.12 ± 0.19	1.30%
2	7	100	200	4–8	3.51 ± 0.14	7.44 ± 0.30	2.35%
3	7	100	200	4–8	3.42 ± 0.11	14.64 ± 0.65	4.62%
3	3	100	200	4–8	2.85 ± 0.07	20.36 ± 0.68	6.42%
3	5	100	200	4–8	3.44 ± 0.13	14.90 ± 0.68	4.70%
3	9	100	200	4–8	3.46 ± 0.14	13.03 ± 0.52	4.11%
3	11	100	200	4–8	3.77 ± 0.19	12.74 ± 0.46	4.02%
3	7	50	200	4–8	4.21 ± 0.14	3.38 ± 0.11	2.13%
3	7	75	200	4–8	4.04 ± 0.18	5.79 ± 0.26	2.43%
3	7	150	200	4–8	3.49 ± 0.14	10.70 ± 0.43	2.25%
3	7	100	150	4–8	3.41 ± 0.12	16.34 ± 0.59	3.86%
3	7	100	300	4–8	3.51 ± 0.12	12.47 ± 0.42	5.90%
3	7	100	400	4–8	3.49 ± 0.16	10.69 ± 0.49	6.74%
3	7	100	200	4–12	3.42 ± 0.14	14.37 ± 0.57	4.53%
3	7	100	200	4–14	3.47 ± 0.13	11.66 ± 0.42	3.68%
3	7	100	200	< 5 mm	3.54 ± 0.12	16.11 ± 0.54	5.08%

## References

[1] Prüss-Ustün A; Bartram J; Clasen T; Colford JM Jr.; Cumming O; Curtis V; Bonjour S; Dangour AD; De France J; Fewtrell L; Burden of disease from inadequate water, sanitation and hygiene in low- and middle-income settings: a retrospective analysis of data from 145 countries. Trop. Med. Int. Health 2014, 19, 894–905. 24779548 10.1111/tmi.12329 PMC4255749

[2] Langwig KE; Frick WF; Reynolds R; Parise KL; Drees KP; Hoyt JR; Cheng TL; Kunz TH; Foster JT; Kilpatrick AM Host and pathogen ecology drive the seasonal dynamics of a fungal disease, white-nose syndrome. Proc. R. Soc. B 2015, 282, 20142335. 10.1098/rspb.2014.2335 PMC4286034 25473016

[3] Zyara AM; Torvinen E; Veijalainen AM; Heinonen-Tanski H The effect of UV and combined chlorine/UV treatment on coliphages in drinking water disinfection. Water 2016, 8, 130. 10.2166/wh.2016.144 27441859

[4] Yoon Y; Chung HJ; Di DYW; Dodd MC; Hur HG; Lee Y Inactivation efficiency of plasmid-encoded antibiotic resistance genes during water treatment with chlorine, UV, and UV/H_2_O_2_. Water Res. 2017, 123, 783–793. 28750328 10.1016/j.watres.2017.06.056

[5] Rajab M; Heim C; Letzel T; Drewes JE; Helmreich B Electrochemical disinfection using boron-doped diamond electrode – the synergetic effects of in situ ozone and free chlorine generation. Chemosphere 2015, 121, 47–53. 25434271 10.1016/j.chemosphere.2014.10.075

[6] Malik MA; Hughes D; Heller R; Schoenbach R Surface plasmas versus volume plasma: energy deposition and ozone generation in air and oxygen. Plasma Chem. Plasma Process. 2015, 35, 697–704.

[7] Chen L; Rajic L; Zhao Y; Hetrick K; Hojabri S; Alshawabkeh A; Xue Y; Zhou W Environmental Remediation with Electrochemical Technologies In Kirk-Othmer Encyclopedia of Chemical Technology; John Wiley & Sons, Inc.: New York, NY, USA, 2018; pp. 1–34.

[8] Doederer K; Gernjak W; Weinberg HS; Farré MJ Factors affecting the formation of disinfection by-products during chlorination and chloramination of secondary e uent for the production of high quality recycled water. Water Res. 2014, 48, 218–228. 24095593 10.1016/j.watres.2013.09.034

[9] Liu C; Olivares CI; Pinto AJ; Lauderdale CV; Brown J; Selbes M; Karanfil T The control of disinfection byproducts and their precursors in biologically active filtration processes. Water Res. 2017, 124, 630–653. 28822343 10.1016/j.watres.2017.07.080

[10] Rodriguez RA; Bounty S; Beck S; Chan C; McGuire C; Linden KG Photoreactivation of bacteriophages after UV disinfection: Role of genome structure and impacts of UV source. Water Res. 2014, 55, 143–149. 24607520 10.1016/j.watres.2014.01.065

[11] Song K; Mohseni M; Taghipour F Application of ultraviolet light-emitting diodes (UV-LEDs) for water disinfection: A review. Water Res. 2016, 94, 341–349. 26971809 10.1016/j.watres.2016.03.003

[12] Oguma K; Katayama H; Mitani H; Morita S; Hirata T; Ohgaki S Determination of pyrimidine dimers in *Escherichia coli* and *Cryptosporidium parvum* during UV light inactivation, photoreactivation, and dark repair. Appl. Environ. Microbiol 2001, 67, 4630–4637. 11571166 10.1128/AEM.67.10.4630-4637.2001 PMC93213

[13] Oguma K; Kita R; Sakai H; Murakami M; Takizawa S Application of UV light emitting diodes to batch and flow-through water disinfection system. Desalination 2013, 328, 24–30.

[14] Sanz EN; Davila IS; Balao JAA; Alonso JMQ Modelling of reactivation after UV disinfection: Effect of UV-C dose on subsequent photoreactivation and dark repair. Water Res. 2007, 41, 3141–3151. 17531283 10.1016/j.watres.2007.04.008

[15] Ikai H; Nakamura K; Shirato M; Kanno T; Iwasawa A; Sasaki K; Niwano Y; Kohno M Photolysis of hydrogen peroxide, an effective disinfection system via hydroxyl radical formation. Antimicrob. Agents Chemother. 2010, 54, 5086–5091. 20921319 10.1128/AAC.00751-10 PMC2981275

[16] Mamane H; Shemer H; Linden KG Inactivation of *E. coli*, *B. subtilis* spores, and MS2, T4, and T7 phage using UV/H_2_O_2_ advanced oxidation. J. Hazard. Mater 2007, 146, 479–486. 17532124 10.1016/j.jhazmat.2007.04.050

[17] Cabiscol E; Tamarit J; Ros J Oxidative stress in bacteria and protein damage by reactive oxygen species. Int. Microbiol 2000, 3, 3–8. 10963327

[18] Bai M; Zhang Z; Xue X; Yang X; Hua L; Fan D Killing effects of hydroxyl radical on algae and bacteria in ship’s ballast water and on their cell morphology. Plasma Chem. Plasma Process. 2010, 30, 831–840.

[19] Hydrogen Peroxide Price. Available online: https://www.kemcore.com/hydrogen-peroxide-50.html (accessed on 12 April 2019).

[20] Qiang Z; Chang JH; Huang CP Electrochemical generation of hydrogen peroxide from dissolved oxygen in acidic solutions. Water Res. 2002, 36, 85–94. 11766820 10.1016/s0043-1354(01)00235-4

[21] Panizza M; Cerisola G Removal of organic pollutants from industrial wastewater by electrogenerated Fenton’s reagent. Water Res. 2001, 35, 3987–3992. 12230183 10.1016/s0043-1354(01)00135-x

[22] Brillas E; Sirés I; Oturan MA Electro-Fenton process and related electrochemical technologies based on Fenton’s reaction chemistry. Chem. Rev 2009, 109, 6570–6631. 19839579 10.1021/cr900136g

[23] Zhou L; Zhou M; Hu Z; Bi Z; Serrano KG Chemically modified graphite felt as an efficient cathode in electro-Fenton for p-nitrophenol degradation. Electrochim. Acta 2014, 140, 376–383.

[24] Zhang X; Huang Y; Chen X; Gao Q; Zhang W Nitrogen-doped carbon nanotubes based on melamine-formaldehyde resin as highly efficient catalyst for oxygen reduction reaction. J. Colloid Interface Sci. 2018, 509, 1–9. 28881199 10.1016/j.jcis.2017.08.096

[25] Alexeyeva N; Shulga E; Kisand V; Kink I; Tammeveski K Electroreduction of oxygen on nitrogen-doped carbon nanotube modified glassy carbon electrodes in acid and alkaline solutions. J. Electroanal. Chem 2010, 648, 169–175.

[26] Dabrowski A; Podkościelny P; Hubicki Z; Barczak M Adsorption of phenolic compounds by activated carbon - a critical review. Chemosphere 2005, 58, 1049–1070. 15664613 10.1016/j.chemosphere.2004.09.067

[27] Xiong Y; He C; Karlsson HT; Zhu X Performance of three-phase three-dimensional electrode reactor for the reduction of COD in simulated wastewater-containing phenol. Chemosphere 2003, 50, 131–136. 12656238 10.1016/s0045-6535(02)00609-4

[28] Zhou W; Rajic L; Chen L; Kou K; Ding Y; Meng X; Wang Y; Mulaw B; Gao J; Qin Y; Activated carbon as effective cathode material in iron-free Electro-Fenton process: Integrated H_2_O_2_ electrogeneration, activation, and pollutants adsorption. Electrochim. Acta 2019, 296, 317–326. 30631212 10.1016/j.electacta.2018.11.052 PMC6322679

[29] Lu Z; Chen G; Siahrostami S; Chen Z; Liu K; Xie J; Liao L; Wu T; Lin D; Liu Y; High-efficiency oxygen reduction to hydrogen peroxide catalysed by oxidized carbon materials. Nat. Catal 2018, 1, 156–162.

[30] Zhou W; Ding Y; Gao J; Kou K; Wang Y; Meng X; Wu S; Qin Y Green electrochemical modification of RVC foam electrode and improved H_2_O_2_ electrogeneration by applying pulsed current for pollutant removal. Environ. Sci. Pollut. Res 2018, 25, 6015–6025. 10.1007/s11356-017-0810-8 29238928

[31] Zarei M; Salari D; Niaei A; Khataee A Peroxi-coagulation degradation of C.I. Basic Yellow 2 based on carbon-PTFE and carbon nanotube-PTFE electrodes as cathode. Electrochim. Acta 2009, 54, 6651–6660.

[32] Yu F; Zhou M; Zhou L; Peng R A novel electro-Fenton process with H_2_O_2_ generation in a rotating disk reactor for organic pollutant degradation. Environ. Sci. Technol. Lett 2014, 1, 320–324.

[33] Chance B; Greenstein DS; Roughton FJW The mechanism of catalase action. I. Steady-state analysis. Arch. Biochem. Biophys 1952, 37, 301–321. 14953443 10.1016/0003-9861(52)90194-x

[34] Zhou X; Mopper K Determination of photochemically produced hydroxyl radicals in seawater and freshwater. Mar. Chem 1990, 30, 71–88.

[35] Stadtman ER; Levine RL Free radical-mediated oxidation of free amino acids and amino acid residues in proteins. Amino Acids 2003, 25, 207–218. 14661084 10.1007/s00726-003-0011-2

[36] Booth IR Regulation of cytoplasmic pH in bacteria. Microbiol. Rev 1985, 49, 359–378. 3912654 10.1128/mr.49.4.359-378.1985 PMC373043

[37] Stadtman ER Metal ion-catalyzed oxidation of proteins: biochemical mechanism and biological consequences. Free Radic. Biol. Med 1990, 9, 315–325. 2283087 10.1016/0891-5849(90)90006-5

[38] Cho M; Lee Y; Chung H; Yoon J Inactivation of *Escherichia coli* by photochemical reaction of ferrioxalate at slightly acidic and near-neutral pHs. Appl. Environ. Microbiol 2004, 70, 1129–1134. 14766597 10.1128/AEM.70.2.1129-1134.2004 PMC348899

[39] Glaze WH; Kang JW; Chapin DH The Chemistry of Water Treatment Processes Involving Ozone, Hydrogen Peroxide and Ultraviolet Radiation. Ozone Sci. Eng 1987, 9, 335–352.

[40] Kumar A; Bisht BS; Joshi VD; Singh AK; Talwar A Physical, chemical and bacteriological study of water from rivers of Uttarakhand. J. Hum. Ecol 2010, 32, 169–173.

[41] Hulicova-Jurcakova D; Seredych M; Lu GQ; Bandosz TJ Combined effect of nitrogen- and oxygen-containing functional groups of microporous activated carbon on its electrochemical performance in supercapacitors. Adv. Funct. Mater 2009, 19, 438–447.

[42] Piddock LJV Clinically relevant chromosomally encoded multidrug resistance e ux pumps in bacteria. Clin. Microbiol. Rev 2006, 19, 382–402. 16614254 10.1128/CMR.19.2.382-402.2006 PMC1471989

[43] Ochman H; Lawrence JG; Groisman EA Lateral gene transfer and the nature of bacterial innovation. Nature 2000, 405, 299–304. 10830951 10.1038/35012500

[44] Rizzo L; Manaia C; Merlin C; Schwartz T; Dagot C; Ploy MC; Michael I; Fatta-Kassinos D Urban wastewater treatment plants as hotspots for antibiotic resistant bacteria and genes spread into the environment: A review. Sci. Total Environ. 2013, 447, 345–360. 23396083 10.1016/j.scitotenv.2013.01.032

[45] GAC Price. Available online: https://www.prnewswire.com/news-releases/global-and-china-activated-carbon-market-2017-2021-−−6-foreign-and-19-chinese-activated-carbon-enterprises-300502107.html (accessed on 12 April 2019).

[46] Schröder E; Thomauske K; Weber C; Hornung A; Tumiatti V Experiments on the generation of activated carbon from biomass. J. Anal. Appl. Pyrolysis 2007, 79, 106–111.

[47] Dias JM; Alvim-Ferraz MCM; Almeida MF; Rivera-Utrilla J; Sánchez-Polo M Waste materials for activated carbon preparation and its use in aqueous-phase treatment: A review. J. Environ. Manag 2007, 85, 833–846. 10.1016/j.jenvman.2007.07.031 17884280

[48] Krembs FJ; Siegrist RL; Crimi ML; Furrer RF; Petri BG ISCO for groundwater remediation: analysis of field applications and performance. Ground Water Monit. Remediat 2010, 30, 42–53.

[49] Thomson NR; Hood ED; Farquhar GJ Permanganate treatment of an emplaced DNAPL source. Ground Water Monit. Remediat 2007, 27, 74–85.

[50] Chen L; Tang M; Chen C; Chen M; Luo K; Xu J; Zhou D; Wu F Efficient bacterial inactivation by transition metal catalyzed auto-oxidation of sulfite. Environ. Sci. Technol 2017, 51, 12663–12671. 28990766 10.1021/acs.est.7b03705

[51] Chen L; Li Z; Chen M Facile production of silver-reduced graphene oxide nanocomposite with highly effective antibacterial performance. J. Environ. Chem. Eng 2019, 7, 103160.

[52] Chen L; Peng Y; Tang M; Wu F Comment on “Combination of cupric ion with hydroxylamine and hydrogen peroxide for the control of bacterial biofilms on RO membranes by Hye-Jin Lee, Hyung-Eun Kim, Changha Lee [Water Research 110, 2017, 83–90]”. Water Res. 2017, 118, 289–290. 27998786 10.1016/j.watres.2016.12.014

[53] Eisenberg G Colorimetric determination of hydrogen peroxide. Ind. Eng. Chem. Anal. Ed 1943, 15, 327–328.

[54] Ashton L; Buxton GV; Stuart CR Temperature dependence of the rate of reaction of OH with some aromatic compounds in aqueous solution. Evidence for the formation of a ˇ-complex intermediate? J. Chem. Soc. Faraday Trans. 1995, 91, 1631–1633.

