# Supplementary material for: Activated Carbon as a Cathode for Water Disinfection through the Electro-Fenton Process
Source: Catalysts. Author manuscript; Available in PMC 2020 Mar 9. (PMC7062377; doi:10.3390/catal9070601)
Supplement: supple [file NIHMS1061026-supplement-supple.pdf]

Supplementary material for

Activated Carbon as a Cathode for Water Disinfection through the Electro-Fenton Process

Long Chen, Ameet Pinto and Akram N. Alshawabkeh *

Department of Civil and Environmental Engineering, Northeastern University, Boston, MA 02115, USA
* Correspondence: a.alshawabkeh@northeastern.edu Tel.: +1-617-373-3994

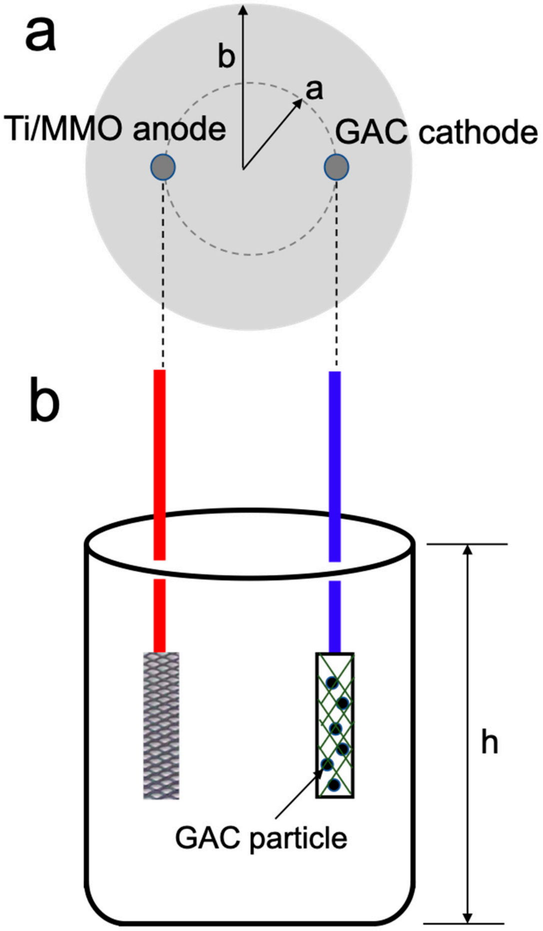

Figure S1. Schematic representation of used reactor at (**a**) top view and (**b**) side view. *a*, 2.5 cm; *b*, 3.5 cm; *h*, 7 cm.

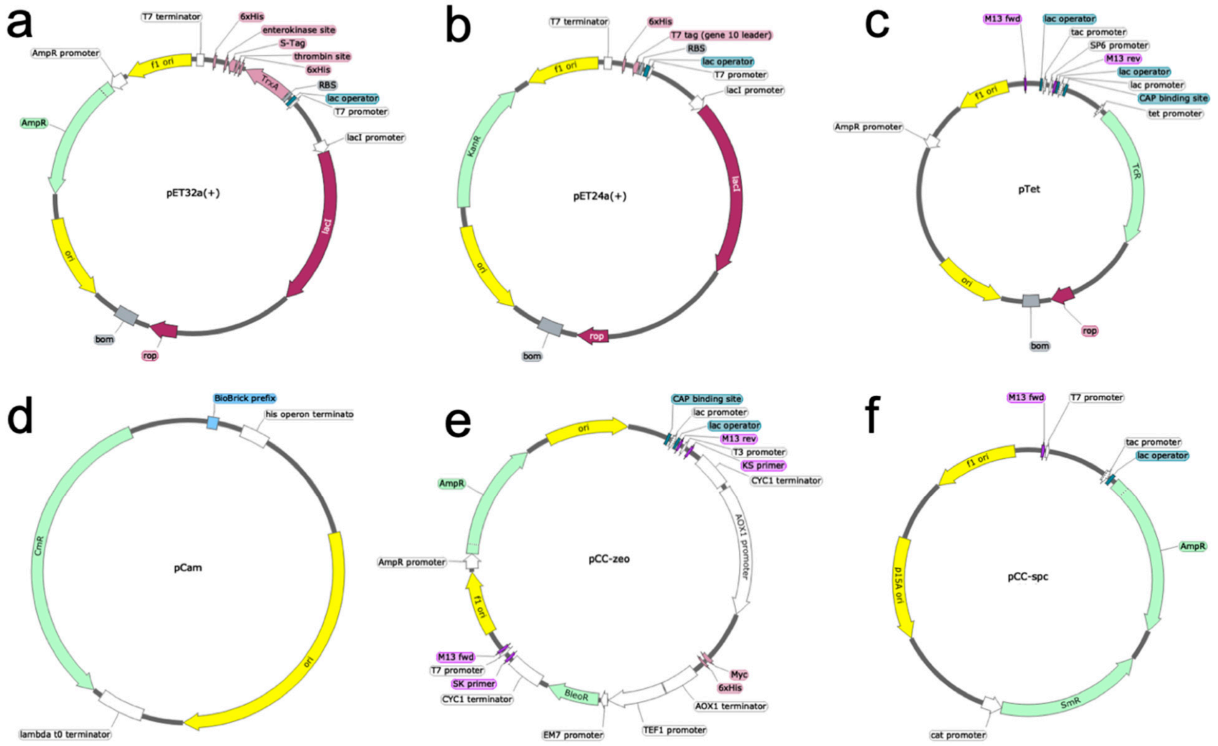

Figure S2. Maps of used plasmids in this study to endow corresponding antibiotic resistance.

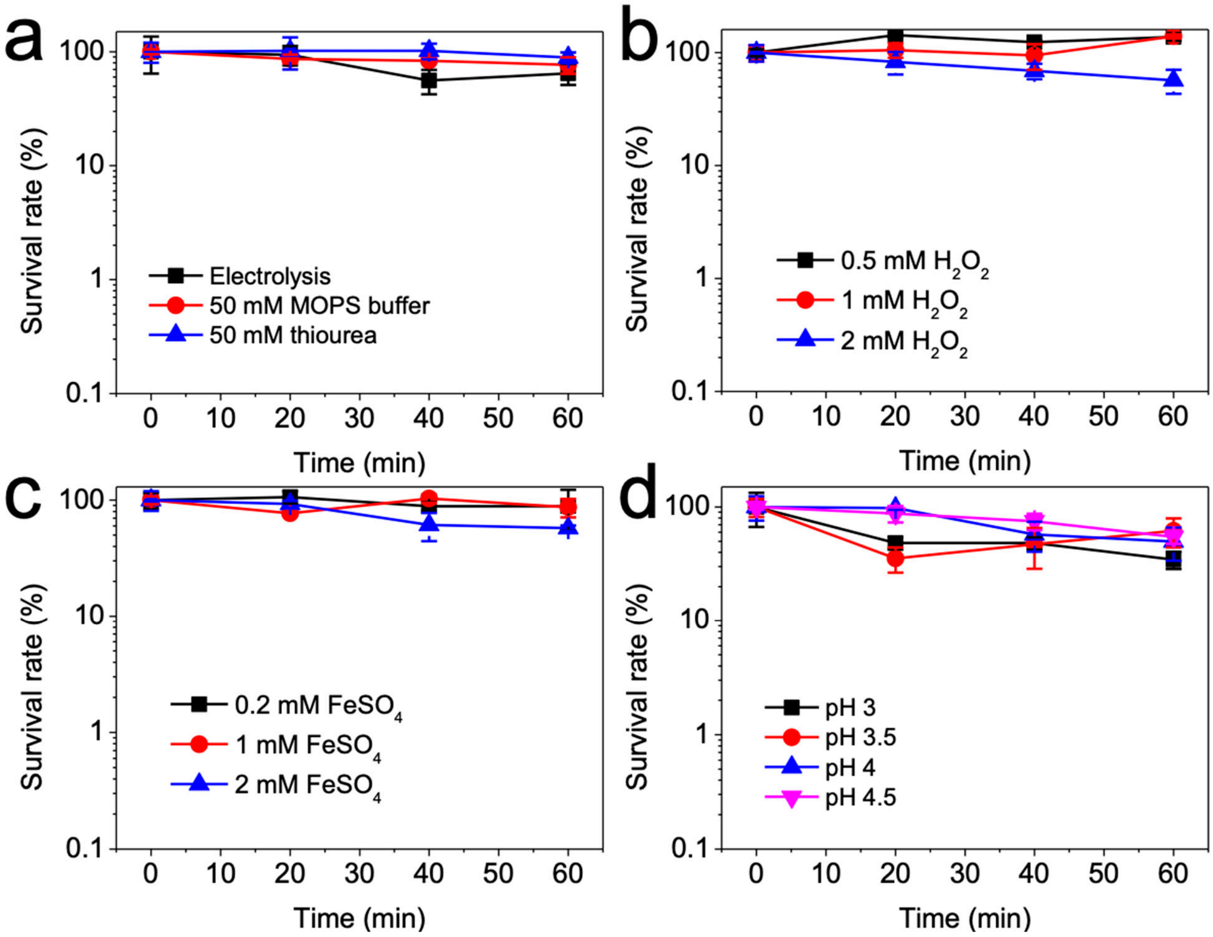

Figure S3. *E. coli* inactivation by (**a**) electrolysis, MOPS buffer or thiourea, (**b**) hydrogen peroxide, (**c**) iron ions, and (**d**) acidic pH.

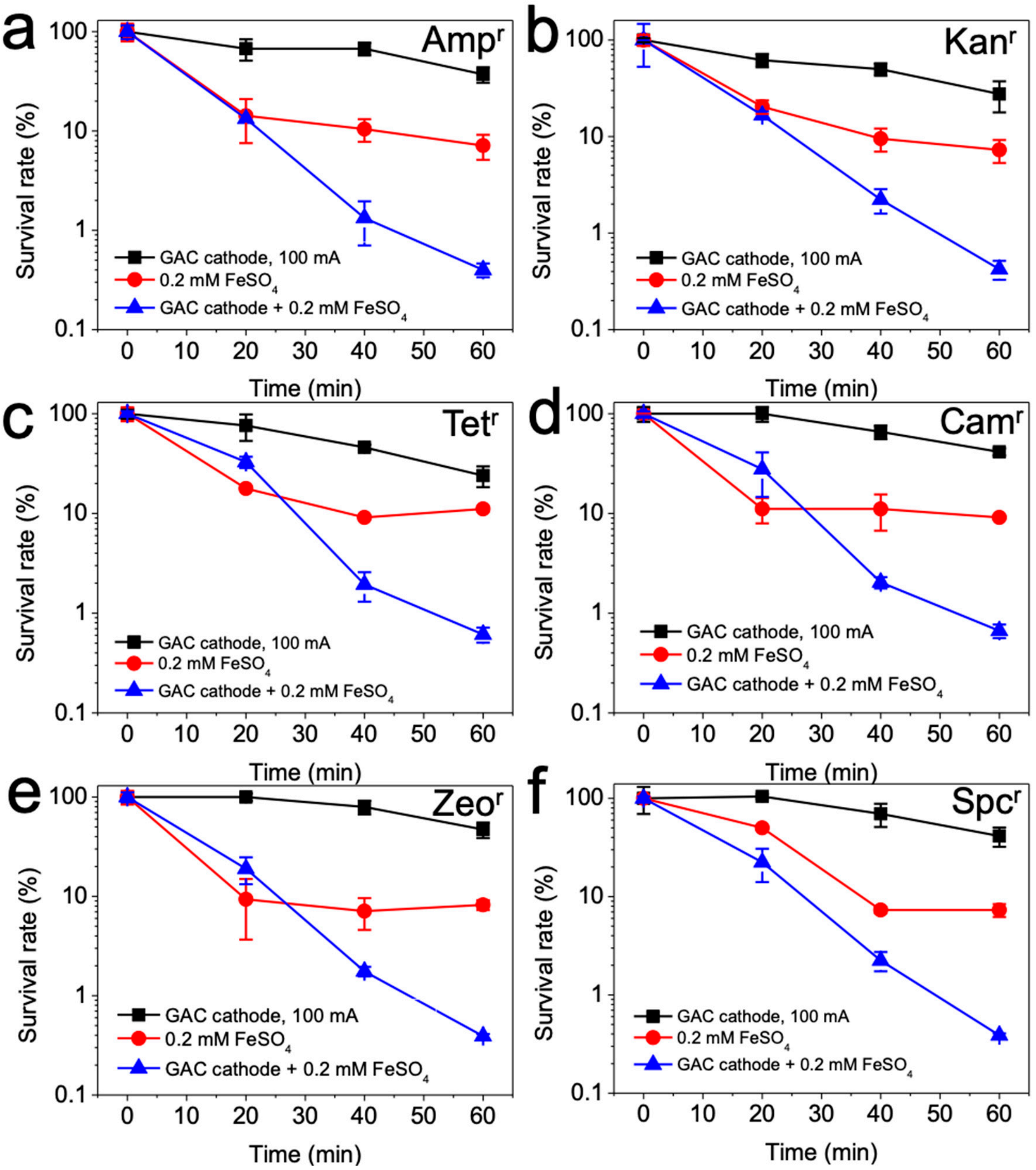

Figure S4. Electro-Fenton process disinfection performance towards *E. coli* of different antibiotic-resistances including control experiments during 60 min. Conditions: 3 g GAC as cathode under 100 mA current, 0.2 mM $FeSO_4$, 10^8 CFU/mL *E. coli*, pH_{ini} 7. Amp^r, ampicillin-resistant; Kan^r, kanamycin-resistant; Tet^r, tetracycline-resistant; Cam^r, chloramphenicol-resistant; Zeo^r, zeocyin-resistant; Spc^r, spectinomycin-resistant.
